# Omalizumab is effective in the preseasonal treatment of seasonal allergic rhinitis

**DOI:** 10.1002/clt2.12094

**Published:** 2022-01-04

**Authors:** Yuan Zhang, Lin Xi, Yunbo Gao, Yanran Huang, Feifei Cao, Wei Xiong, Chengshuo Wang, Luo Zhang

**Affiliations:** ^1^ Department of Allergy Beijing TongRen Hospital Capital Medical University Beijing China; ^2^ Beijing Key Laboratory of Nasal Diseases Beijing Institute of Otolaryngology Beijing China; ^3^ Research Unit of Diagnosis and Treatment of Chronic Nasal Diseases Chinese Academy of Medical Sciences Beijing China; ^4^ Department of Otolaryngology Head and Neck Surgery Beijing TongRen Hospital Capital Medical University Beijing China

**Keywords:** omalizumab, pollen season, preseasonal treatment, seasonal allergic rhinitis

## Abstract

**Background:**

To date no study has evaluated the efficacy of preseasonal omalizumab therapy with cost effective dose and at appropriate time point compared with standard medication in seasonal allergic rhinitis (SAR) patients.

**Methods:**

This was a prospective randomized controlled open‐label single‐centre trial. 32 SAR patients were randomized to receive a single injection of omalizumab 300‐mg approximately two weeks before start of the pollen period (PP) or medication therapy. All patients completed daily questionnaires; recording symptoms, medication use and quality of life (QoL) throughout the observation period. The primary efficacy parameter was the mean daily Combined Symptom and Medication Score (CSMS).

**Results:**

Preseasonal omalizumab significantly reduced the changes of mean daily CSMS of nose during the PP (*p* < 0.001), peak pollen period (PPP) and PP after PPP (PPP‐PP) (*p* = 0.002) and Post‐PP (*p* = 0.009) compared to standard medication. The proportion of allergy symptoms‐relieving medication‐free days during PPP‐PP was also significantly higher in preseasonal omalizumab‐treated group (76.2(16.7‐98.8))% than in medication‐treated group (19.0(0‐71.4))% (*p* = 0.030). Omalizumab could achieve the same nasal symptom control during the entire pollen season and better eye symptoms relieving results in PP (*p* = 0.046) and PPP‐PP (*p* = 0.004) than medication treatment. Significantly greater improvement in QoL was also obtained with omalizumab‐pretreatment during the PP (*p* = 0.037) and PPP‐PP (*p* = 0.004).

**Conclusions:**

Administration of a single injection of 300 mg omalizumab two weeks before start of the pollen season achieves better overall control of symptoms and QoL, with significantly reduced allergy symptoms‐relieving medication usage, compared with standard pharmacotherapy in SAR patients.

## 1 INTRODUCTION

Allergic rhinitis (AR) is a chronic inflammatory disease caused when inhaled allergens contact the nasal mucosa and induce an immunoglobulin E (anti‐immunoglobulin E(IgE))‐mediated response; which results in the symptoms of nasal itching, rhinorrhea, sneezing, nasal obstruction, ocular pruritus, redness and/or lacrimation. Allergic rhinitis can be classified into seasonal AR (seasonal allergic rhinitis (SAR)) and perennial AR (PAR) and outdoor pollens are major causes for SAR. It has been demonstrated that sensitization to common pollens such as artemisia and ragweed tends to lead to persistent and moderate/severe AR^1^ and the disease control is not satisfactory.^2^ A recent study involving 6043 subjects from the grasslands of Northern China has demonstrated that 18.5% of these subjects had pollen‐induced AR (PiAR) based on allergen tests,^3^ indicating a large population of SAR patients. In patients who are allergic to pollen, the nasal mucosa priming phenomenon starts and the symptoms of AR present almost as soon as pollination begins; becoming more severe when pollen concentrations are highest until the end of the pollen season.^4^ Extra attention needs to be directed towards management of SAR.

Selection of pharmacotherapy for patients with AR aims to control the disease and depends on many factors such as symptom severity and self‐management strategies.^5^ It has been pointed that guidelines recommended standard therapies are not sufficiently followed because they are not close enough to patients' needs and probably do not reflect real life.^5^ Actually, an early study investigating AR patients in UK general practice has reported that only 27% of the patients used standard medication involving both oral antihistamines and intranasal corticosteroids (INS) regularly, and 62% of these subjects described their symptom control as partial or poor.^6^ More recent evidence also suggests that adherence to treatment is fairly low in allergic diseases and asthma,^7^, ^8^ suggesting that novel treatments or treatment strategies may be needed to improve adherence and management of patients whose symptoms are inadequately controlled with standard medication care. In this regard, a recombinant humanized anti‐immunoglobulin E (IgE) antibody (omalizumab), which mainly blocks the binding of IgE to high‐affinity receptors (FceRI) on effector cells and thereby prevents the activation of the IgE‐mediated disease, has been shown safe and effective in the treatment of patients with moderate‐to‐severe allergic asthma,^9^ refractory chronic spontaneous urticaria,^10^ AR^11^, ^12^ and chronic rhinosinusitis with nasal polyps.^13^

To date no study has evaluated the efficacy of preseasonal omalizumab therapy with cost effective dose and at appropriate time point compared with standard therapy in SAR patients. Thus, the aim of the present study was to compare the efficacy of a single dose of omalizumab treatment with standard medication administered approximately 2 weeks prior to the pollen period (PP) in controlling symptoms and medication use during the autumn fall season in Chinese patients with SAR.

## 2 METHODS

### 2.1 Ethics statement

The study (NCT04489121) was approved by the medical ethics committee of Beijing TongRen Hospital, and written informed consent was obtained from each patient before participation.

### 2.2 Study design

**FIGURE 1:**
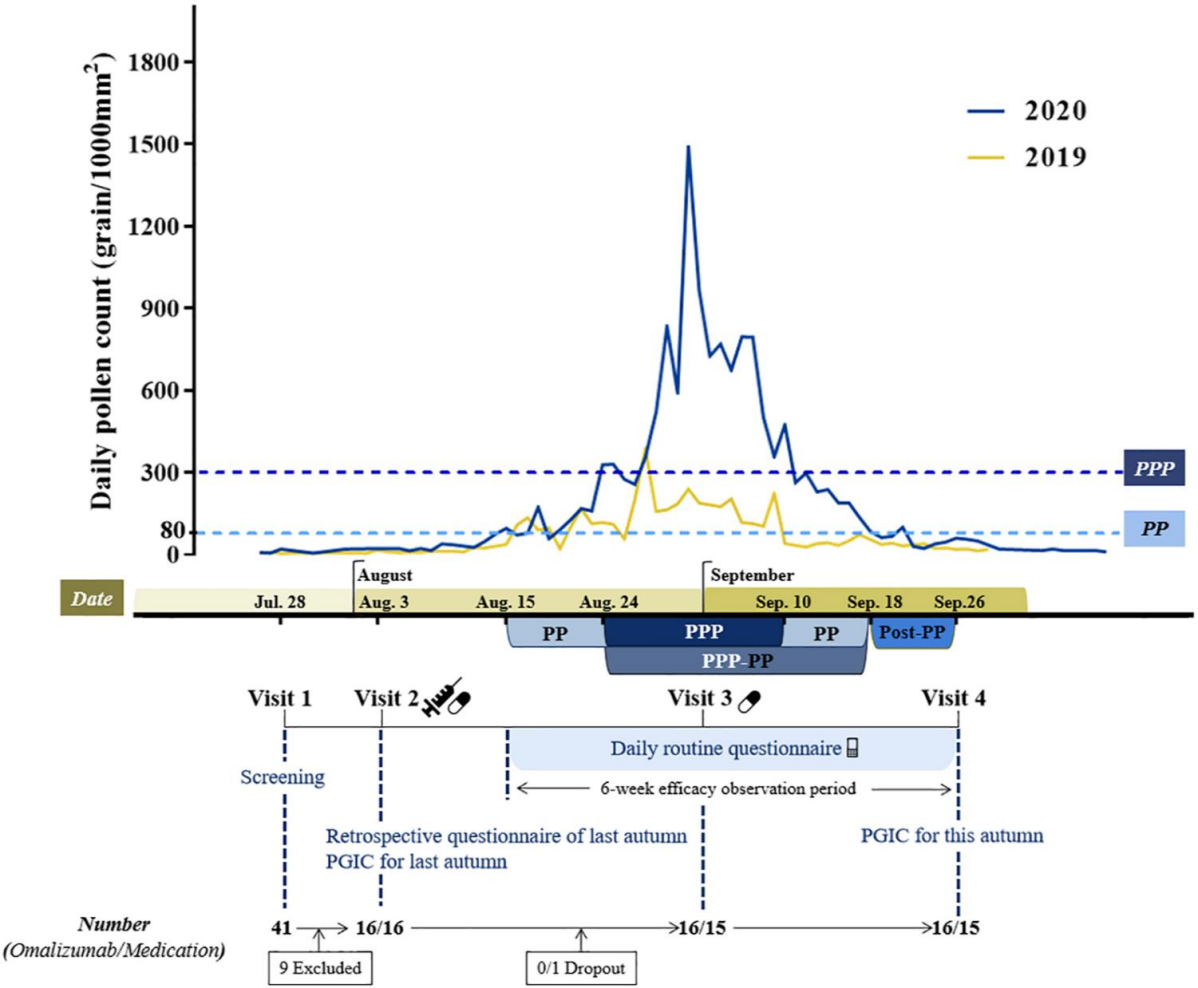
Study design. PGIC: Patient global impression of change; PP: pollen period; PPP‐PP: peak pollen period (PPP) and PP after PPP; Post‐PP: post pollen period

This was a prospective randomized controlled open‐label single‐centre clinical trial undertaken in the Department of Allergy, Beijing TongRen Hospital during the PP from July 2020 to September 2020. The study involved four visits as follows: visit 1 (screening), visit 2 (randomization, initiation of omalizumab injection and distribution of allergy‐relieving medications), visit 3 (supplementary distribution of medications) and visit 4(completion of the study) (Figure 1).

Subjects were recruited in July 2020 from a cohort of outpatients who had previously visited the departmental clinic between July and October in 2018 and 2019; and for whom symptoms, medication scores and results of allergen tests were recorded in the department's database. The diagnosis of AR was based on the latest AR and its Impact on Asthma (ARIA) guideline,^14^ and patients were eligible for recruitment to the study if they satisfied the following inclusion criteria: (1) aged 18 to 60 years; (2) lived in Beijing for several years and would be residing and working in Beijing during the study period; (3) had a clinical history of autumn SAR for at least two years, with/without conjunctivitis; (4) had demonstrated symptoms scores of ≥2 points for two or more nasal symptoms (sneezing, rhinorrhea, nasal itching and nasal obstruction) and for at least one conjunctival symptoms (ocular itching/grittiness/redness and ocular tearing) during July—October in the last two years; (5) demonstrated sensitization to at least one of the main autumn pollens; including artemisia, ragweed and humulus scandens; as confirmed by the presence of specific immunoglobulin E (sIgE; ≥0.7 kU_A_/L) using ImmunoCAP system (Pharmacia, Uppsala, Sweden); (6) demonstrated baseline total IgE ≥30 kU/L.

Patients were excluded from the study if they met any of the following criteria: (1) PAR symptoms and sensitization to indoor allergens including dust mites, mold and animal hairs; (2) any nasal condition that could confound the results of the study including nonallergic rhinitis, chronic rhinosinusitis with/without polyps; (3) comorbid asthma or atopic dermatitis; (4) treated with a systemic glucocorticoid within 4 weeks or oral antihistamine and intranasal corticosteroid within 2 weeks prior to recruitment; (5) treated with allergen specific immunotherapy for pollens within last 5 years; (6) any kind of surgery within 4 weeks prior to recruitment; (7) participation in any clinical study within the 3 months prior to recruitment; (8) pregnant, breast‐feeding/sexually active women of childbearing potential; (9) patients at risk of non‐compliance; (10) Patients with immunologic suppression, diabetes mellitus, autonomic neuropathy, coronary heart disease or hypertension.

Eligible patients were randomized in ratio of 1:1 to receive either omalizumab or the control standard medication according to a computer‐generated randomization code. Omalizumab was administered about 2 weeks before the start of the autumn PP as a single 300‐mg subcutaneous injection by a designated nurse with no other role in the trial, based on the findings of Casale and colleagues.^12^ The following drugs were permitted as allergy symptoms‐relieving medications according to the actual needs in both groups: Clarityne (tablet), Budesonide (nasal spray) and Patanol (eye drop), which were distributed free of charge to patients at visit 2 and visit 3. Concomitant use of any other agent apart from the above mentioned an allergy symptoms‐relieving medication was prohibited.

All patients included in the study completed daily questionnaires; recording symptoms, medication use and quality of life (QoL); over a period of 6 weeks from the first day of the PP to one week after the end of PP. Considering the continuous influence of peak pollen period (PPP) on the patients, the efficiency of treatment was presented as three phases separately, that is, PP (data of days in PP before PPP), PPP‐PP (data of days in PPP, and PP after PPP) and Post‐PP (data of days within one week after PP ending). Retrospective routine questionnaires and Patient Global Impression of Change (PGIC) of AR exacerbations in last autumn were completed in Visit 2. Patient Global Impression of Change for the autumn of 2020 was calculated at Visit 4. All the questionnaires were completed by electronic version pushed through WeChat. Safety of treatment was also assessed according to the adverse events (AEs) profile, which was monitored and recorded in daily questionnaire throughout the study.

Daily concentrations of pollen were also recorded over the course of the study.

### 2.3 Definition of pollen phase

Data of daily total pollen concentration; expressed as grains per 1000 mm^2^ at 16 stationary monitoring stations distributed in the urban and suburban districts of Beijing; were provided by Beijing Meteorological Bureau (http://bjweather.iyuebo.com/weather.php?a=chart2). Based on the findings of a previous study, which suggested that the effect of pollen on nasal and ocular symptoms is maximal and plateaus after a saturation point of 80 and 90 grains/m^3^, respectively,^15^ we defined the autumn PP as the period between the first day when the total daily pollen count was ≥80 total pollen/1000 mm^2^ to the first day when the total daily pollen count was <80 total pollen/1000 mm^2^. Similarly, the PPP was defined as the period between the first and last three consecutive days with ≥300 total pollen/1000 mm^2^ each day.^11^, ^16^

The date of about two weeks before the start of the autumn PP for 2020, and thus the date for initiation of treatment, was estimated based on data of daily pollen concentrations in Beijing in recent years.^16^ For the purpose of the present study, it was estimated that the date for the start of the autumn PP in Beijing would fall between mid to late August, and thus the initiation of treatment was arranged in early August (August 3–4). Once data provided by Meteorological Bureau demonstrated the total autumn pollen count was <80 total pollen/1000 mm^2^ each day for three days, this suggested that the PP had ended and the study was concluded 1 week later (defined as Post‐PP in this study). Based on daily pollen data as shown in Figure 1, the PP for 2020 lasted from August 15 to September 18, and the PPP from August 24 to September 10. The Post‐PP period was during September 19 to September 26.

### 2.4 Efficacy and safety assessments

The primary efficacy parameter was the mean daily Combined Symptom and Medication Score (CSMS) for the nose (CSMS‐nose).^17^ Secondary efficacy measures included CSMS for eyes (CSMS‐eye), average daily medication score (MS), proportion of allergy symptoms‐relieving medication‐free days, total nasal symptom score (TNSS), total eye symptom score (TESS), mini Rhinoconjunctivitis Quality of Life Questionnaire (RQLQ) score, and PGIC.

The routine daily questionnaires mainly involved three aspects of the effect of treatment on symptoms, medication usage and QoL using the mini RQLQ.^18^ The subjective assessment of AR symptoms was generally based on the patient scores for four nasal symptoms (sneezing, rhinorrhea, nasal itching and nasal obstruction), and two ocular symptoms (ocular itching/grittiness/redness and ocular tearing); scored range of 0 (not at all) to 3 (severe). Total nasal symptom score and TESS were assessed as the sum of the scores for the four nasal symptoms and the two eye symptoms, respectively.

Medication score was calculated according to the use of specific drug/s on a three‐point scale; with 1 = oral and/or topical (eyes or nose) nonsedative H1 antihistamines (H1A); 2 = INS with/without H1A; and 3 = oral corticosteroids with or without INS, with or without H1A.

The daily CSMS was calculated as follows: (total symptom scores ranging from 0 to 12 for nose or 0 to 6 for eye)/number of symptoms + MS. CSMSs concerning nose as well as eye were assessed.

Mini RQLQ, which contains 14 questions scored between 0 and 6 for activities, sleep, practical problems, nose symptoms, eye symptoms and emotional function,^19^ was used to assess the QoL.

Patient Global Impression of Change was evaluated according to a five‐point scale of 0‐4; with 0 = symptoms were aggravated; 1 = no control over symptoms; 2 = minor control over symptoms; 3 = substantial control over symptoms; and 4 = total control over symptoms.^20^

### 2.5 Statistical analysis

The patients were assigned to the omalizumab group or to the control standard medication group at a 1: 1 ratio. The sample size in this study was calculated according to two aspects of data using PASS11 (NCSS Corp) before the start of the study. Firstly, we referred to studies investigating the treatment efficacy of omalizumab in Japanese cedar pollen‐induced SAR,^11^, ^21^ which used the method of non‐inferiority test to evaluate the efficacy of omalizumab and control group, and calculated the sample size by setting a non‐inferiority margin of 0.5, the power (1 − *β*) of 0.8 and two sided α of 0.05. The true difference of CSMS between the two groups at the pollen peak reported in the studies was about 1.93 and standard deviation about 2.05. Secondly, in order to reduce the impact of changes in population, environment and other factors, we also carried out a preliminary experiment to compare the efficacy of omalizumab (*n* = 8) and conventional medications (*n* = 8) in the treatment of SAR patients in last autumn. This study demonstrated a true difference of CSMS in PPP‐PP was 0.93 and standard deviation was 1.33. Based on a combination of data from the studies in Japanese cedar pollen‐induced SAR^10^, ^21^ and the preliminary study, in the current study we have set true difference as 0.9 and standard deviation as 1.3 as the parameters for calculating the sample size, and found that at least 12 subjects would be required in each group. Based on this finding and allowing for the potential loss to follow‐up, we recruited an additional 20% of subjects, the sample size finally included 16 patients in the omalizumab group and 15 patients in the control group. According to the data observed at the pollen peak, it was found that the true difference was 1.37 and SD was 1.61, and thus the calculated (1 − β) was 0.93, suggesting that the current sample size had sufficient power in this study.

Statistical analyses were performed using the Statistical Product and Service Solutions 23.0 software (IBM Corp.). Data were assessed for normality and equal variation, and results were expressed as mean ± standard error of mean (SEM). Chi square test was used to analyze classified data. Student *t* test or Mann–Whitney *U* test was used to analyze the differences between groups. Pairwise treatment comparisons were obtained from a 2‐way analysis of variance (ANOVA) and he changes in clinical parameters were evaluated by repeated‐measures ANOVA analyses. *p* < 0.05 was considered to be statistically significant.

## 3 RESULTS

The recruitment started from July 28, 2020 as planned in clinical trial registration (https://www.clinicaltrials.gov/). A total of 41 patients with autumn SAR were screened, of whom 32 patients were eligible and were randomized in equal numbers to receive omalizumab or standard medication. One patient in the medication treatment group discontinued because of noncompliance of daily questionnaire, and eventually 16 patients in omalizumab group and 15 patients in the standard medication treatment group completed study eventually (Supporting information Figure S1).

### 3.1 Demographic and clinical characteristics

The demographic and clinical characteristics of the patients in the two study groups are demonstrated in Table 1. The two groups were not significantly different with regard to age, gender, weight, duration of autumn SAR, smoking and drinking history, AR family history as well as the status of concomitant allergic conjunctivitis. Similarly, both groups were comparable with regard to the baseline serum total IgE and serum Phadiatop sIgE levels(*p* > 0.05), as well as TNSS, TESS, MS, RQLQ and PGIC scores recorded during the last autumn (*p* > 0.05). Moreover, the baseline regarding TNSS, TESS, MS as well as RQLQ in this autumn between groups also presented as comparable (*p* > 0.05).

**TABLE 1:** Demographic and clinical characteristics of study population

Characteristic	Omalizumab (*n* = 16)	Medication treatment (*n* = 15)	*p* value
Age (years), mean ± SEM	34.75 ± 1.75	36.93 ± 2.55	0.478
Sex, female/male, No (%)	10(62.5)/6(37.5)	10(66.7)/5(33.3)	0.709
Weight (Kg), mean ± SEM	68.06 ± 10.82	63.11 ± 8.63	0.181
Duration of AR(years), mean ± SEM	8.81 ± 0.97	8.14 ± 1.32	0.682
Smoking history, N	1	0	0.367
Drinking history, N	1	1	0.574
Family history of AR, N	8	6	0.534
History of allergic conjunctivitis, N	6	9	0.272
Baseline serum total IgE level (kU/L), mean ± SEM	381.24 ± 85.32	256.69 ± 88.70	0.321
Baseline serum Phadiatop sIgE (kU_A_/L), mean ± SEM	8.81 ± 0.97	8.14 ± 1.33	0.130
Baseline sensitization profile (Mono/Poly), N	14*/2	14*/1	0.583
Last autumn			
TNSS, mean ± SEM	8.69 ± 0.92	10.13 ± 0.53	0.187
TESS, mean ± SEM	3.38 ± 0.52	4.53 ± 0.42	0.099
MS, mean ± SEM	1.31 ± 0.29	1.40 ± 0.25	0.826
CSMS‐nose, mean ± SEM	3.48 ± 0.43	3.93 ± 0.29	0.403
CSMS‐eyes, mean ± SEM	3.00 ± 0.45	3.67 ± 0.30	0.223
RQLQ score, mean ± SEM	47.81 ± 5.80	52.33 ± 3.18	0.502
PGIC score, median (IQR)	2(2‐3)	2(2‐3)	0.511
Baseline in this autumn			
TNSS, mean ± SEM	1.44 ± 0.24	1.57 ± 0.29	0.732
TESS, mean ± SEM	0.38 ± 0.12	0.53 ± 0.13	0.377
MS, mean ± SEM	0.44 ± 0.13	0.40 ± 0.13	0.839
CSMS‐nose, mean ± SEM	0.68 ± 0.16	0.85 ± 0.14	0.453
CSMS‐eyes, mean ± SEM	0.63 ± 0.16	0.67 ± 0.14	0.848
RQLQ, mean ± SEM	5.65 ± 0.89	7.42 ± 0.57	0.107

*Note*: *mono‐sensitization to Artemesia.

Abbreviations: AR, allergic rhinitis; CSMS, Combined Symptom and Medication Score; IQR, interquartile range; MS, medication score; PGIC, Patient Global Impression of Change; RQLQ, Rhinoconjunctivitis Quality of Life Questionnaire; SE, standard error of mean; TESS, total eye symptom score; TNSS, total nasal symptom score.

In omalizumab group, among the 16 individuals administered a subcutaneous dose of 300 mg omalizumab, seven patients were considered to be adequately dosed as calculated on the basis of baseline serum total IgE level and weight.

### 3.2 Efficacy

**FIGURE 2:**
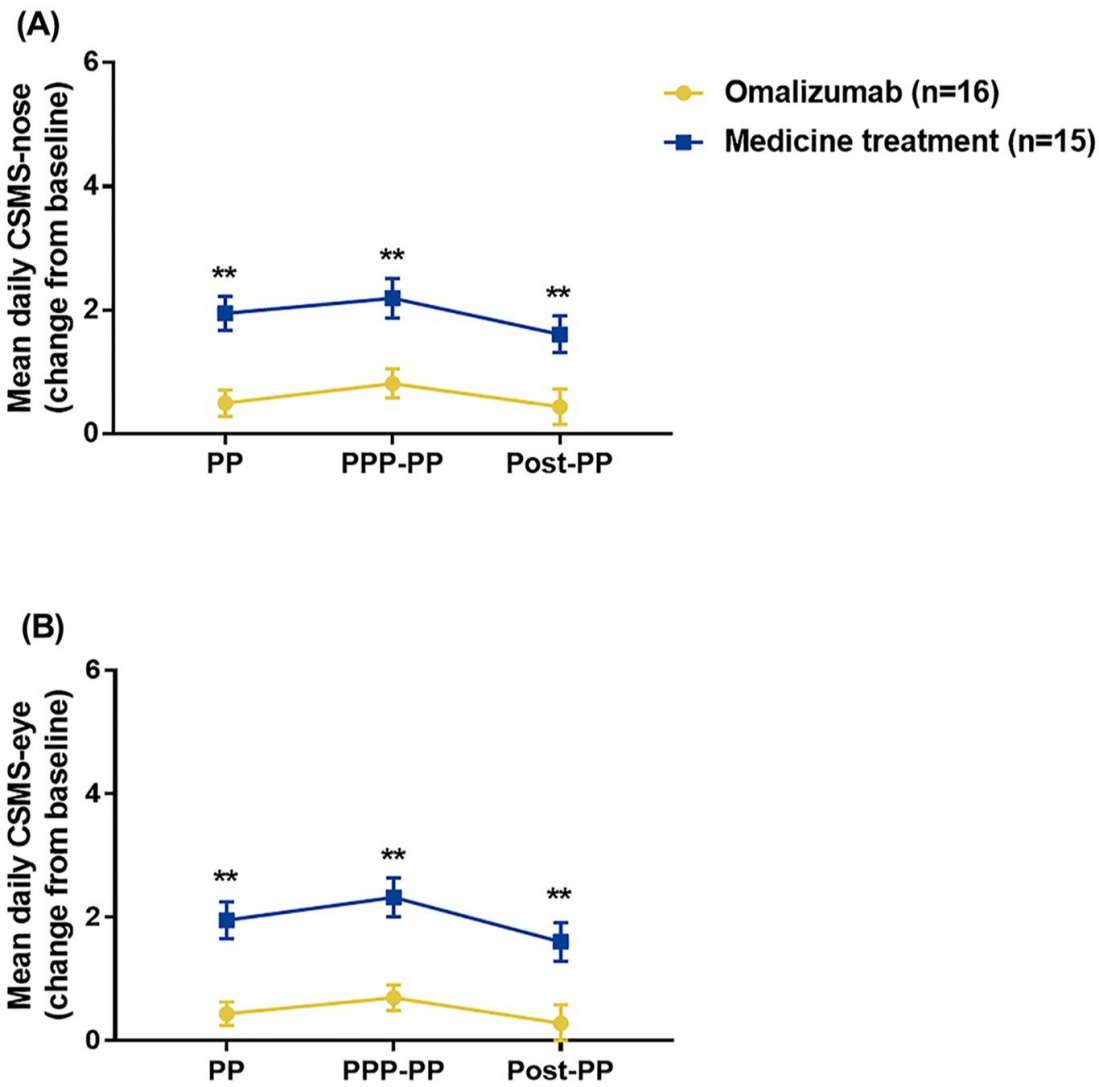
Comparison of mean daily Combined Symptom and Medication Score (CSMS) between omalizumab pretreatment and standard medication treatment groups during pollen period PP, PPP‐PP and Post‐PPP of autumn 2020. CSMS: combined symptoms medication score (MS). PP: pollen period; PPP‐PP: peak pollen period (PPP) and PP after PPP; Post‐PP: post pollen period. *p < 0.05; **p < 0.01

The primary efficacy parameter was CSMS‐nose. Omalizumab pretreatment for the current autumn 2020 pollen season significantly decreased changes of mean daily CSMS‐nose scores during the PP (*p* < 0.001), PPP‐PP (*p* = 0.002) and Post‐PP (*p* = 0.009) compared with standard medication (Figure 2A). Moreover, among the patients receiving omalizumab, there was no significant difference in changes of CSMS‐nose scores during the different periods of pollen season (*P*_*PP*_ = 0.399; *P*_*PPP‐PP*_ = 0.407; *P*_*Post‐PP*_ = 0.408) between the patients who were considered to have been sufficiently dosed compared with those considered to be insufficiently dosed with omalizumab. In addition, as the omalizumab group had comparable baseline total IgE levels and symptoms during the last autumn compared with medication group, regression analyses with an interaction term were performed to evaluate the effect on the efficacy. We found that there was no association between baseline total IgE levels and CSMS improvement during the entire autumn pollen season as shown in Supporting information Figure 2 (Omalizumab: *P*_*PP*_ = 0.645; *P*_*PP‐PPP*_ = 0.900; *P*_*P‐PPP*_ = 0.991, Medicine treatment: *P*_*PP*_ = 0.870; *P*_*PP‐PPP*_ = 0.338; *P*_*P‐PPP*_ = 0.706). Similarly, regression analysis of data for symptoms during the last autumn and CSMS improvement also demonstrated that there was no association between symptoms during the last autumn and CSMS improvement during the entire autumn pollen season as shown in Supporting information Figure 3 (Omalizumab: *P*_*PP*_ = 0.062; *P*_*PP‐PPP*_ = 0.341; *P*_*P‐PPP*_ = 0.403, Medicine treatment: *P*_*PP*_ = 0.598; *P*_*PP‐PPP*_ = 0.860; *P*_*P‐PPP*_ = 0.731).

**FIGURE 3:**
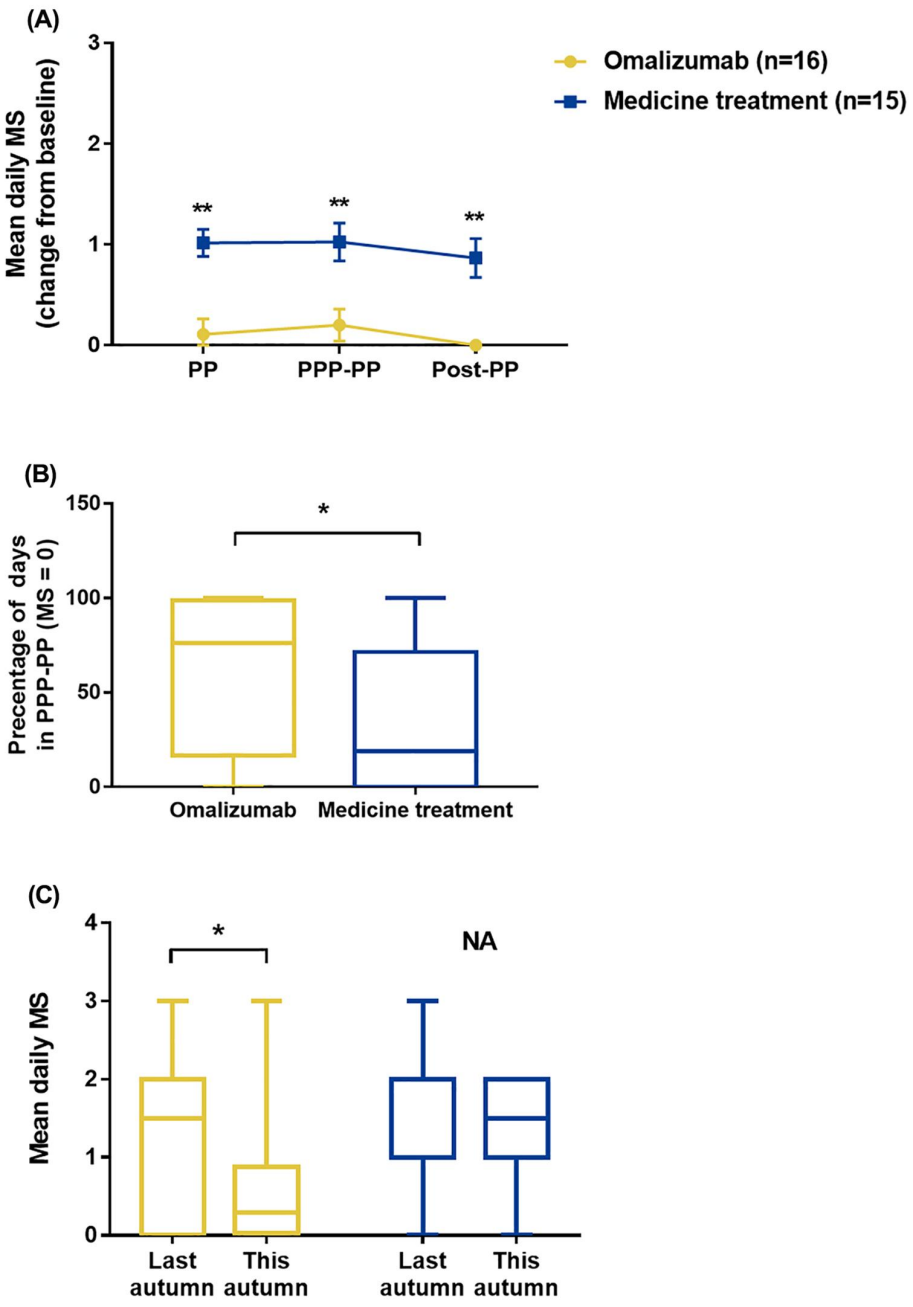
Comparison of medication usage between omalizumab pretreatment and standard medication groups during pollen period (PP), PPP‐PP and Post‐PPP of autumn 2020. MS: medication score. PP: pollen period; PPP‐PP: peak pollen period (PPP) and PP after PPP; Post‐PP: post pollen period. *p < 0.05; **p < 0.01

Assessment of the effect of omalizumab pretreatment on the secondary efficacy measure during the 2020 pollen season demonstrated that omalizumab also significantly decreased the changes of mean daily CSMS‐eye scores during PP (*p* < 0.001), PPP‐PP (*p* < 0.001) and Post‐PP (*p* = 0.003), compared with medication treatment (Figure 2B). Similarly, omalizumab pretreatment significantly decreased the changes of average daily MS during PP (*p* = 0.001), PPP‐PP (*p* = 0.003) and Post‐PP (*p* < 0.001), compared with medication therapy (Figure 3A). Moreover, the proportion of allergy symptoms‐relieving medications‐free days during PPP‐PP was significantly higher in omalizumab pretreatment group (76.2(16.7–98.8)% than in standard medication treatment group (19.0(0‐71.4))% (*p* = 0.030) (Figure 3B). Additionally, compared with the retrospective MS from autumn of 2019, the average daily MS in PP of autumn 2020 was significantly lower in omalizumab pretreated patients (*p* = 0.044) but not in standard medication treated patients (*p* = 0.958) (Figure 3C).

**FIGURE 4:**
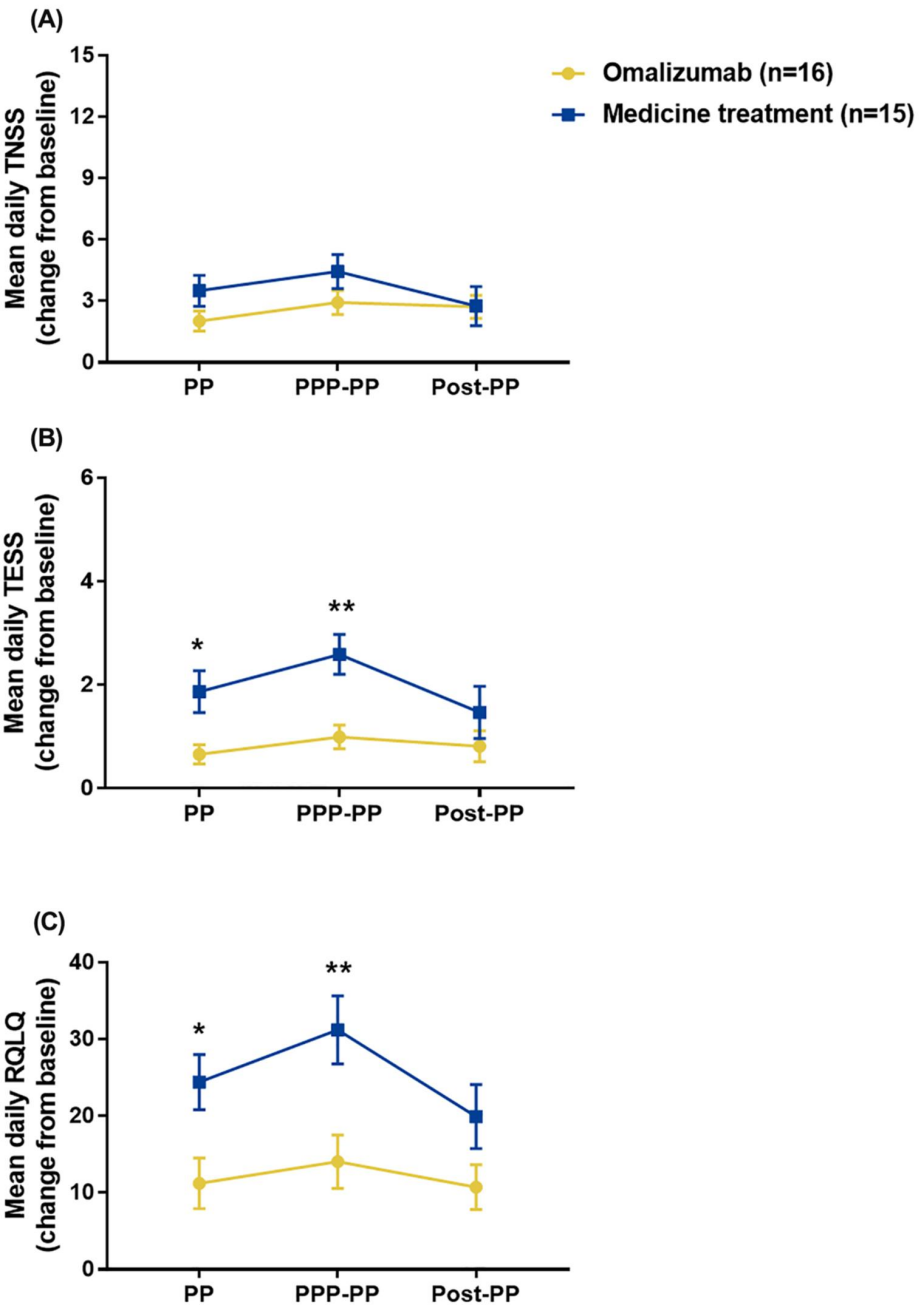
Comparison of nasal and eye symptoms severity and quality of life (QoL) between omalizumab pretreatment and convention medication treatment groups during pollen period (PP), PPP‐PP and Post‐PPP of autumn 2020. TNSS: total nasal symptom score; TESS: total eye symptom score; MS: medication score; RQLQ: Rhinoconjunctivitis QoL Questionnaire. PP: pollen period; PPP‐PP: peak pollen period (PPP) and PP after PPP; Post‐PP: post pollen period. *p < 0.05; **p < 0.01

Concerning the symptoms scores, despite significantly reduced allergy symptoms‐relieving drug usage (Figure 3A), omalizumab pretreatment achieved the same level of total nasal symptoms control, assessed as TNSS, as standard medication treatment during PP (*p* = 0.123), PPP‐PP (*p* = 0.117) as well as Post‐PP (*p* = 0.874) of the current pollen season (Figure 4A). Assessment of TESS, however, demonstrated that, omalizumab pretreatment led to significantly better control of eye symptoms compared to standard medication treatment during PP (*p* = 0.046) and PPP‐PP (*p* = 0.004) (Figure 4B). Furthermore, patients in omalizumab pretreatment group achieved significantly better QoL scores compared with patients in the standard medicine group over the entire pollen season (*P*_*PP*_ = 0.037; *P*_*PPP‐PP*_ = 0.004) (Figure 4C).

**FIGURE 5:**
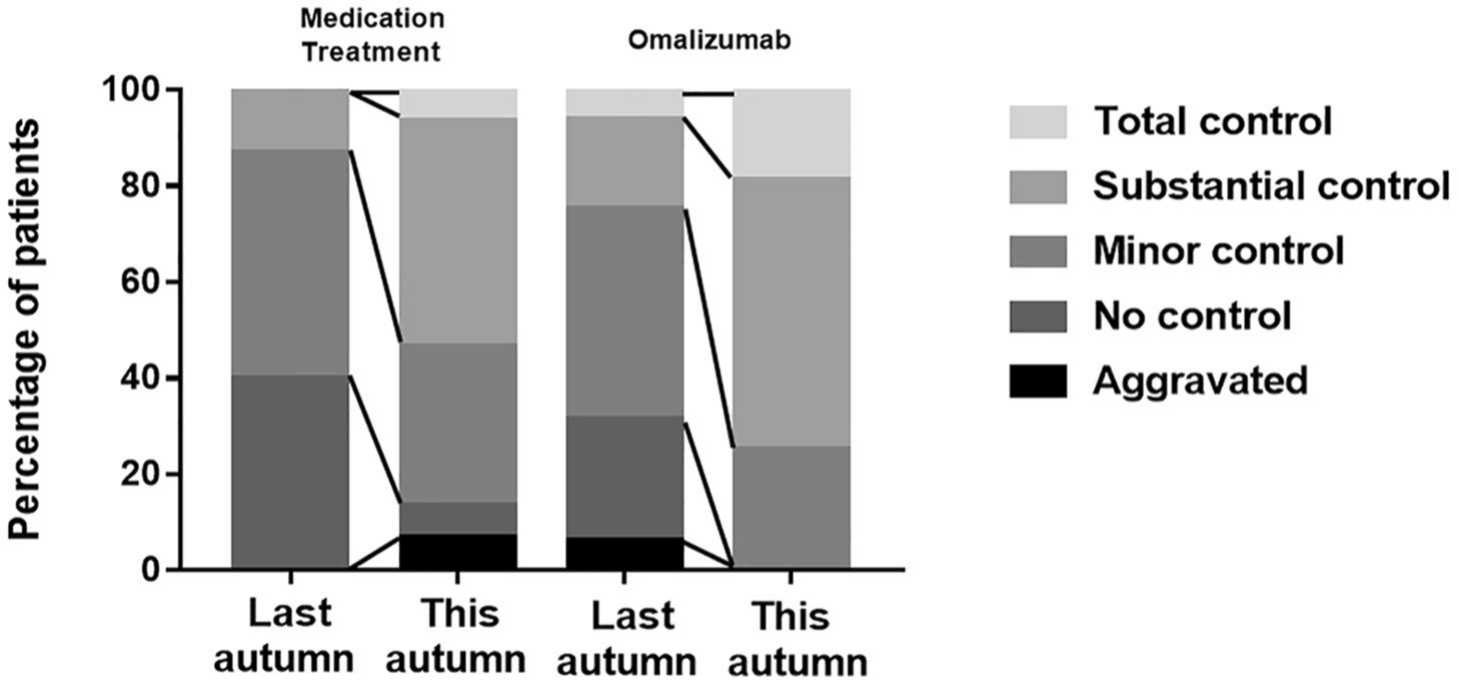
Comparison of Patient Global Impression of Change (PGIC) between omalizumab pretreatment and convention medication treatment groups during pollen period (PP) in autumn 2019 and 2020. PGIC: Patient Global Impression of Change; PP: pollen period

The findings for patient clinical overall impression rating are presented graphically as Figure 5. Treatment effectiveness was globally rated as total and substantial control by 18.75% and 56.25% of the patients, respectively, in omalizumab pretreatment group and by 6.67% and 46.67% of the patients, respectively, in standard medication group. Comparison of global assessments for patients in the omalizumab pretreated group demonstrated that 31.25% of the patients had indicated that their symptoms were not controlled or even aggravated following treatment during the 2019 pollen season compared with 0% of the patients following omalizumab pretreatment during the 2020 pollen season. Similarly, the percentage of patients who considered their symptoms to be totally controlled increased from 6.25% in 2019%, to 18.75% in 2020. In contrast, 13.33% patients receiving standard medication indicated that their AR symptoms were not controlled or worsened and 6.67% of the patients indicated that their symptoms were completely controlled compared to the previous pollen season.

### 3.3 AEs

Only one of the 16 patients (6.25%) in the omalizumab pretreatment group experienced local AEs, which presented as local itching and wind masses at the injection site and subsided within half an hour after the injection. No systemic AEs were observed among any of the patients receiving omalizumab.

## 4 DISCUSSION

This prospective randomized controlled open‐label single‐centre study, has shown that adding omalizumab 300‐mg to a preseasonal regimen of guidelines‐based therapy for autumnal SAR in adults, 2 weeks before the autumn pollen season, can significantly decrease allergy symptoms‐relieving medication use and concurrently achieve the same level of nasal symptoms control, greater eye symptoms control and significant improvements in QoL of patients, as standard therapy with medication. To our knowledge this is the first study to demonstrate that only a single injection of preseasonal omalizumab in a clear time point of administration combined with standard therapy during the pollen season may provide better overall control in the management of AR over the course of the entire pollen season than use of standard therapy for SAR. Furthermore, these findings set precedence for routine use of this novel seasonal approach in the treatment of SAR in the future.

The majority of SAR patients undergo treatment involving mainly guideline–based pharmacotherapy with antihistamines and corticosteroids, protective measures to reduce pollen inhalation or their adhesion to the conjunctiva and nasal mucosa, and allergen immunotherapy (allergen immunotherapy (AIT)). Although AIT is the only aetiological treatment for AR and can significantly reduce SAR symptoms and medication usage,^16^, ^22^ it is relatively difficult for patients to adhere due to the at least three years' treatment duration and high cost. An observational study involving over 12,000 AR patients has recently reported that 69.05% AR patients were also non‐adherent to medications,^8^ indicating that medication‐taking behaviour in a real‐world setting is likely to be different to that in a controlled clinical trial setting. Moreover, up to 35% of patients treated according to guidelines‐based pharmacotherapy have been shown to have uncontrolled symptoms; suggesting a need for optimization of treatment strategies and embracing the principles of precision medicine in chronic airways diseases, in order to achieve a higher level of disease control, and enhance patients' adherence and satisfaction.^23^

anti‐immunoglobulin E and mast cells play a pivotal role in allergic diseases and treatment with omalizumab, has significantly improved control of these diseases and introduced a new era for the management of severe allergic conditions.^24^ Several placebo‐controlled studies of omalizumab in the treatment for AR have confirmed its effectiveness and safety in patients with both SAR and PAR.^11^, ^12^, ^25^ However, due to the relative high cost of omalizumab, a reduced cost of treatment for only the fall season to treat SAR patients with severe symptoms during the onset might be more justifiable compared with treatment for the whole year in PAR individuals. In this respect, studies reported that omalizumab provided clinical benefit in a dose‐dependent fashion in patients with SAR^12^ and significant clinical efficacy in Japanese cedar pollen induced SAR patients.^11^ The common treatment strategy employed in the studies in SAR^11^, ^12^ was that different doses of omalizumab (ranging from 50 to 375 mg) or placebo was administered 3 or 6 times to patients over the course of the entire 12‐week pollen season. One study investigating the effect of preseasonal treatment of omalizumab on preventing fall asthma exacerbations in school children proposed intervention treatments from 4 to 6 weeks before the school start date to 90 days after the school start date, that is, over a period of about 4 months.^26^ In comparison, the current study has investigated a novel treatment strategy involving administration of a single injection of preseasonal omalizumab 300 mg during the pollen season, with the goal of attaining at least equally or possibly greater efficiency of treatment in controlling the symptoms of AR compared with pharmacotherapy and simultaneously minimizing the overall cost of omalizumab. In this regard, the current novel treatment strategy does indeed meet both goals, as indicated by achievement of satisfactory nasal and eye symptoms control and QoL under the premise of significantly reduced allergy symptoms‐relieving medication usage in SAR patients. Moreover, in a real life setting with low adherence to standard pharmacotherapy, the difference of efficacy between the omalizumab and control groups would be probably higher. The “single omalizumab dose” approach ensures that SAR patients are being well covered during the entire season. Of course, the specific performance of a single dose of omalizumab may probably vary among allergen seasons with different lengths.

In addition, a fixed 300 mg dose of omalizumab was chosen here as Casale TB et al. demonstrated that SAR‐specific nasal symptom severity scores and QoL scores were consistently better in patients who received 300 mg of omalizumab than in those who received other dosages or placebo and did not decline during peak season.^12^ Likewise, there was no significant difference between the improvements in symptoms noted in patients considered to have received a sufficient dose of omalizumab compared to patients considered to have received an insufficient dose of omalizumab in present study. CSMS was selected as the primary outcome based on European Academy of Allergy and Clinical Immunology recommendations for the standardization of clinical outcomes in AIT trials for allergic rhinoconjunctivitis.^17^ Although TNSS has often been chosen as the primary endpoint in the majority of the published placebo‐controlled studies of omalizumab in the treatment of AR, the use of concomitant medication has also been considered as an important efficacy parameter in many studies.^11^, ^12^ As allergy symptoms‐relieving medications are allowed to be used throughout the study, any improvement in patient's symptoms by the medication itself should also be taken into account. In this sense, the validated system for a “weighted” CSMS balances out these problems well. Moreover, the principle of CSMS has been found to be associated with a large effect size, thereby leading to a high power to show treatment efficacy.^27^

Treatment with omalizumab in the current study was also initiated before the onset of the autumn season to assess whether blocking IgE binding before the pollen season could reduce SAR symptoms during the entire pollen season. Indeed, an early study by Pipkorn et al.^28^ hypothesized that beginning treatment before symptoms develop might prevent the priming effect of allergic inflammation in the nasal mucosa that lead to increased reactivity to allergen challenge when the seasonal pollen progresses. The theoretical basis of pre‐seasonal treatment approach might also be attributable to dampening of “minimal persistent inflammation (minimal persistent inflammation (MPI))”, which has been shown to be constantly detectable in asymptomatic mite‐sensitized patients continuously exposed to low levels of allergen and elicits a state of heightened sensitivity to subsequent allergen exposure in these patients.^29^ There is evidence that MPI is also present in the nasal passages of asymptomatic subjects with SAR and this is exacerbated by high pollen exposure during the pollen season.^30^, ^31^ Further studies are required to address the specific mechanism/s underlying the effects of omalizumab in the nasal mucosa of patients with SAR during pollen exposure.

Last but not least, the present study is limited in some aspects. In particular, no objective and/or laboratory test was investigated as an outcome, and as this was not a placebo‐controlled study and involved small size of study population, the true benefit of a single dose of preseasonal omalizumab in the management of the symptoms of SAR during the pollen season cannot be evaluated fully. Consequently, this is also likely to reduce the level of evidence of this study. Although the findings from the present study that an effective preventative strategy for autumn SAR can be achieved with targeted preseasonal omalizumab treatment suggest a paradigm shift for how to manage SAR patients, further confirmation is needed from multicentre, randomised, double‐blinded, placebo‐controlled studies involving larger well‐characterised study populations with multiple sensitization profiles, with respect to both phenotypes and endotypes of SAR patients, who are most likely to benefit from such a novel approach. Moreover, the effects of omalizumab need to be investigated over a wider dose range for optimal effects. Similarly, the optimal period for pretreatment with omalizumab prior to the start of the pollen season needs to be addressed.

## CONFLICT OF INTEREST

All authors declare no financial or commercial conflicts of interest.

## Supporting information

Supplementary Material

Supplementary Material

Supplementary Material

Supplementary Material
