# Supplementary material for: Omalizumab is effective in the preseasonal treatment of seasonal allergic rhinitis
Source: Clin Transl Allergy. 2022 Jan 4;12(1):e12094. doi: 10.1002/clt2.12094 (PMC8727318; doi:10.1002/clt2.12094)
Supplement: Supplementary file 1 — Supplementary Material [file CLT2-12-e12094-s004.docx]

**Supporting information
Figure 1.** Patient disposition and flow diagram. RQLQ: Rhinoconjunctivitis quality of life questionnaire; PGIC: Patient global impression of change.

**Figure 2.** Regression analyses of baseline total IgE levels on CSMS improvement during the entire autumn pollen season.

CSMS: combined symptoms medication score. PP: pollen period; PPP-PP: peak pollen period (PPP) and PP after PPP; Post-PP: post pollen period.

**Figure 2.** Regression analyses of symptoms during the last autumn on CSMS improvement during the entire autumn pollen season.

CSMS: combined symptoms medication score. PP: pollen period; PPP-PP: peak pollen period (PPP) and PP after PPP; Post-PP: post pollen period.
