# Supplementary figures and images for: Omalizumab is effective in the preseasonal treatment of seasonal allergic rhinitis
Source: Clin Transl Allergy. 2022 Jan 4;12(1):e12094. doi: 10.1002/clt2.12094 (PMC8727318; doi:10.1002/clt2.12094)

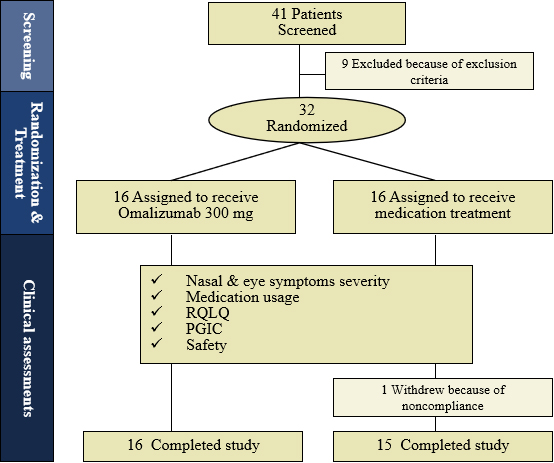

Supplement: Supplementary file 2 — Supplementary Material [file CLT2-12-e12094-s001.jpg]

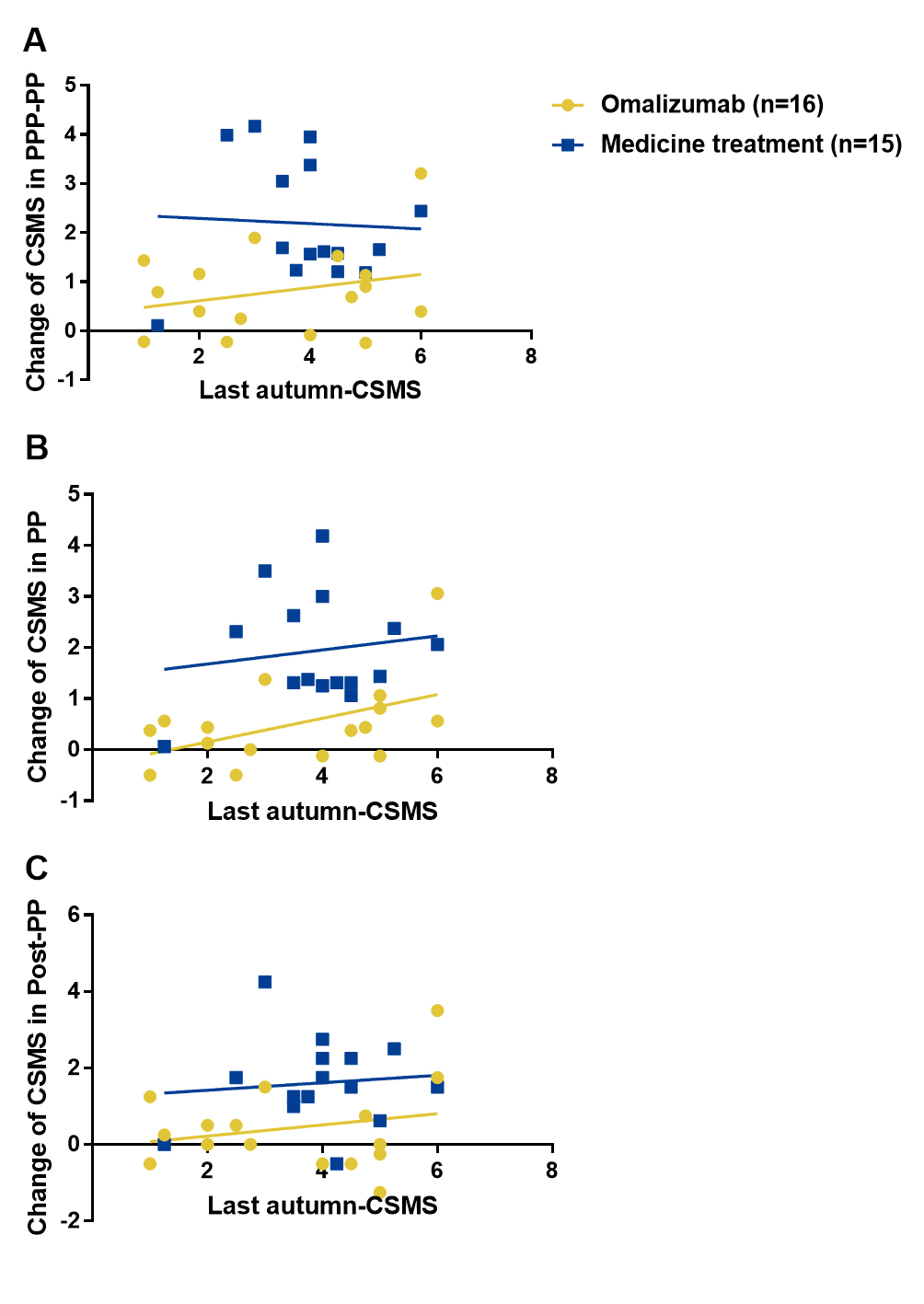

Supplement: Supplementary file 3 — Supplementary Material [file CLT2-12-e12094-s003.jpg]

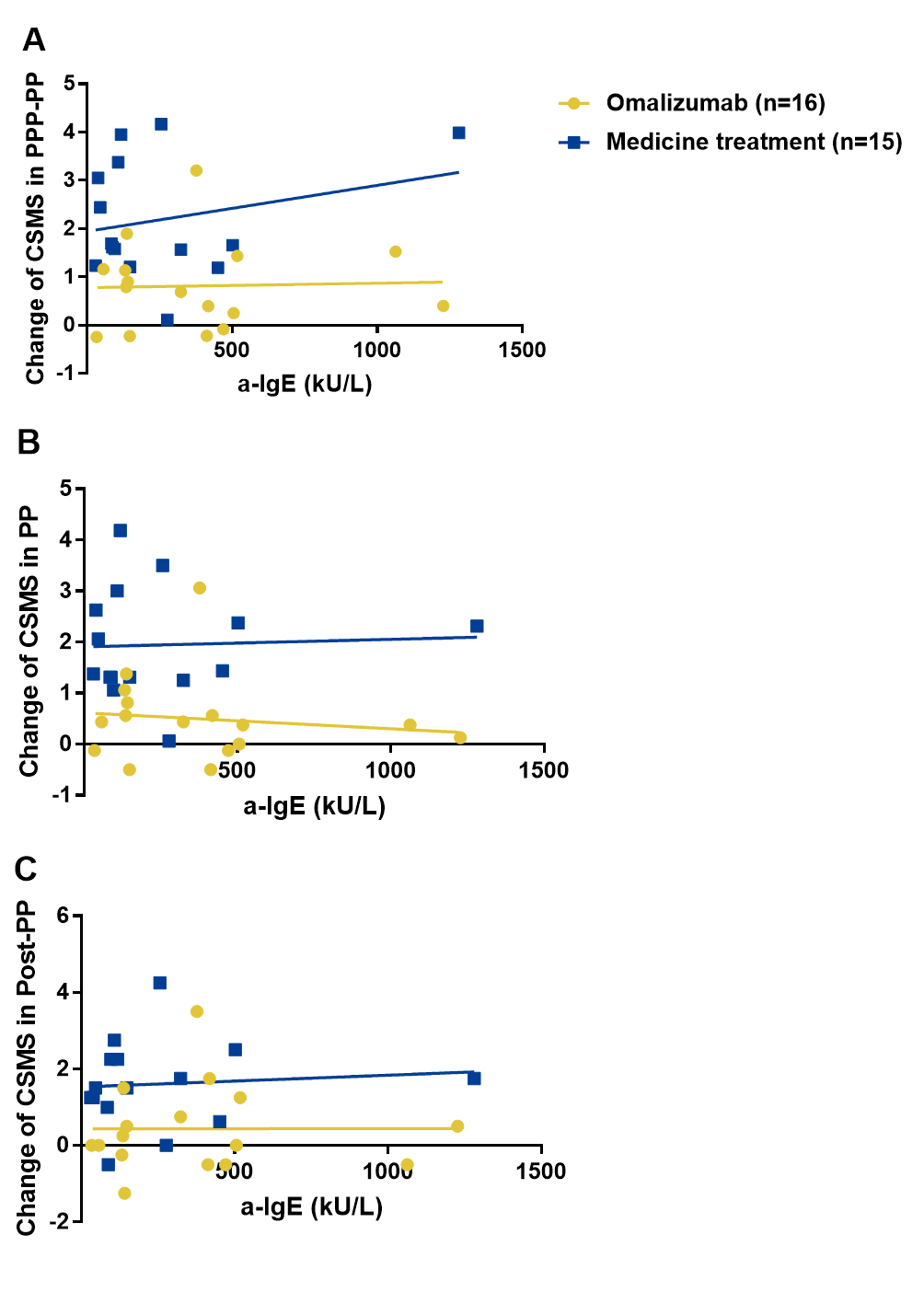

Supplement: Supplementary file 4 — Supplementary Material [file CLT2-12-e12094-s002.jpg]
